# Deficiency of AMPK in CD8^+^ T cells suppresses their anti-tumor function by inducing protein phosphatase-mediated cell death

**DOI:** 10.18632/oncotarget.3501

**Published:** 2015-03-09

**Authors:** Enyu Rao, Yuwen Zhang, Ganqian Zhu, Jiaqing Hao, Xuan-Mai T. Persson, Nejat K. Egilmez, Jill Suttles, Bing Li

**Affiliations:** ^1^ The Hormel Institute, University of Minnesota, Austin, MN, USA; ^2^ Center for Clinical and Translational Science Metabolomics Core, Mayo Clinic, Rochester, MN, USA; ^3^ Department of Microbiology and Immunology, University of Louisville, Louisville, KY, USA

**Keywords:** AMPK, CD8^+^ T cells, T cell survival, anti-tumor function

## Abstract

A number of studies have linked AMPK, a major metabolic sensor coordinating of multiple cellular functions, to tumor development and progression. However, the exact role of AMPK in tumor development is still controversial. Here we report that activation of AMPK promotes survival and anti-tumor function of T cells, in particular CD8^+^ T cells, resulting in superior tumor suppression *in vivo*. While AMPK expression is dispensable for T cell development, genetic deletion of AMPK promotes T cell death during *in vitro* activation and *in vivo* tumor development. Moreover, we demonstrate that protein phosphatases are the key mediators of AMPK-dependent effects on T cell death, and inhibition of phosphatase activity by okadaic acid successfully restores T cell survival and function. Altogether, our data suggest a novel mechanism by which AMPK regulates protein phosphatase activity in control of survival and function of CD8^+^ T cells, thereby enhancing their role in tumor immunosurveillance.

## INTRODUCTION

AMP-activated protein kinase (AMPK) is an evolutionarily conserved serine/threonine protein kinase that has been identified as a master sensor in maintaining cellular energy homeostasis [1]. As a heterotrimeric enzyme, AMPK consists of a catalytic subunit (α) and two regulatory subunits (β and γ), in which phosphorylation of the threonine 172 residue of the α-subunit is crucial for the activation of AMPK. It has been shown that at least three kinases function as major upstream activators of AMPK: the liver kinases B1 (LKB1), calmodulin-dependent protein kinase kinases (CAMKKs) and TGF-β-activated kinase 1 (TAK1) [2-4]. LKB1 complex phosphorylates AMPKα in response to elevated AMP/ATP ratio in cells during energy stress, whereas CAMKKs and TAK1 activate AMPKα in response to elevated intracellular Ca^2+^ levels or extracellular TGF-β stimulation, respectively, regardless of the energy status inside cells. In addition, recent studies also identify reactive oxygen species (ROS) as another upstream activator of AMPK [5, 6]. Once AMPK is activated, it promotes ATP production by increasing the activity of proteins involved in catabolic pathways while reducing ATP consumption by switching off anabolic pathways. For example, activated AMPK can regulate fatty acid (FA) metabolism by enhancing FA oxidation while inhibiting FA synthesis through phosphorylation and inactivation of acetyl-CoA carboxylase (ACC) [7]. Thus, AMPK plays a critical role in the regulation of many aspects of cellular metabolism and function, including cell proliferation, survival, autophagy and immune responses [8].

Due to its diverse functions in regulating cellular fundamental activities, AMPK has been proposed to be involved in tumorigenesis and tumor metabolic transformation in many studies. AMPK-activating drugs, such as metformin, have been shown to significantly delay the onset of tumorigenesis and to suppress tumor progression [9, 10]. Activation of AMPK opposes tumor growth by negatively regulating the Warburg effect of tumor cells [11]. Of note, while numerous studies supports the anti-tumor function of AMPK, there is accumulating evidence suggesting a pro-tumorigenic role of AMPK. For example, activation of AMPK promotes tumor cell survival during energy stress [12]. AMPK is also essential for solid tumor formation in different tumor models [13, 14]. Considering the complexity of tumorigenesis involving the interactions of both tumor cell intrinsic factors and extrinsic immunosurveillance, deeper insights into the mechanisms of how AMPK regulates immune cell functions in tumor development will facilitate our understanding of those seemingly contradictory observations.

It has been clear that host immunity is essential in the control of tumor development [15, 16]. Among the components of the immune system, T cells, in particular CD8^+^ T cells, play a critical role in mediating tumor immunosurveillance [17, 18]. CD8^+^ T cells can directly lyse tumor cells by exocytosis of perforins and granzymes [19]. They can also induce tumor cell apoptosis by production of TNF family members, such as FasL, TRAIL [20]. Moreover, tumor-specific CTL produce a large amount of IFNγ, which increases expression of MHC class I molecules and Fas on cancer cells, thus leading to enhanced granule- and death ligand-mediated tumor cytotoxicity [21, 22]. More importantly, emerging evidence substantiates the anti-tumor effect of tumor-infiltrating CD8^+^ T cells as determined by their presence and favorable effect on survival of patients with various types of cancer [23, 24]. As T cell functions are regulated not only by external signals (e.g. TCR stimulation or cytokines), but also by internal metabolic properties [25], emerging evidence has shown that AMPK plays critical roles in T cell metabolism and memory CD8^+^ T cell differentiation [26, 27]. However, AMPK regulates T cell anti-tumor activity remains largely unknown.

In the present study, we generated T cell-specific AMPK knockout mice and demonstrated that deletion of AMPK in T cells accelerates tumor growth in mice. More specifically, the increased tumor growth is mainly mediated by the defective anti-tumor functions of AMPK-deficient CD8^+^ T cells. Deletion of AMPK in CD8^+^ T cells results in decreased production of IFNγ and Granzyme B. Mechanistically, AMPK deficiency in T cells enhances serine/threonine protein phosphatase activity upon activation, thus leading to elevated cell death and impaired anti-tumor functions. Our findings provide evidence to support a novel role of AMPK in the anti-tumor activity of CD8^+^ T cells.

## RESULTS

### Phenotypic analysis of AMPKα1 deficiency in T cells

**Figure 1:**
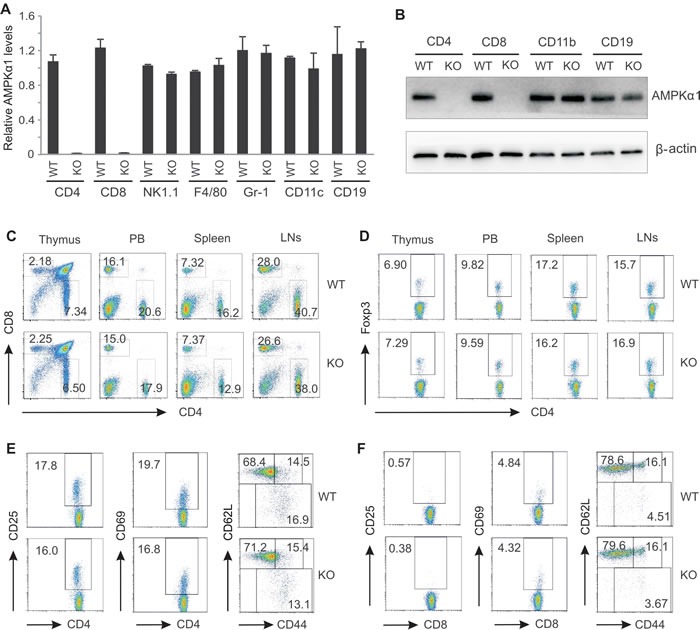
AMPKα1 deficiency has no impact on T cell development Analysis of relative mRNA and protein levels of AMPKα1 in different splenic populations from WT and AMPK KO mice by real-time RT-PCR (A) and western blot (B). C, flow cytometric analysis of CD4^+^ and CD8^+^ T cells in the thymus, peripheral blood (PB), the spleen and lymph nodes (LNs) from WT and KO mice. D, analysis of natural regulatory T cells in the thymus, PB, the spleen and LNs from WT and KO mice by intracellular staining of Foxp3 expression. Phenotypic analysis of the expression of CD25, CD69, CD62L and CD44 on CD4^+^ T cells (E) and on CD8^+^ T cells (F) from the spleen of WT and KO mice by flow cytometric staining.

As a major metabolic sensor, AMPK is ubiquitously expressed in various tissues and cells with different isoforms. The catalytic subunit of AMPKα1 was the predominant isoform in T cells (Supplementary Figure 1A). To explore the function of AMPK in T cells, mice carrying a floxed *Prkaα1* gene were crossed to CD4-cre mice which harboring the recombinase Cre under the control of CD4 promoter to conditionally delete AMPKα1 expression in T cells during the double positive stage of T cell development. The AMPKα1^*fl/fl*^ *Cre*^+^ (KO) and AMPKa1^*fl/fl*^ *Cre*^−^ (WT) littermates were used for the study. Analysis of AMPK expression by real-time PCR confirmed that AMPKα1 was successfully deleted in both CD4^+^ and CD8^+^ T cells in AMPKα1 KO mice. In contrast, AMPKα1 expression in other immune populations, including CD11b^+^ cells, F4/80^+^ macrophages, CD11c^+^ DC cells, CD19^+^ B cells, Gr-1^+^ neutrophils, NK1.1^+^ NK cells, was not affected (Figure 1A). Consistent with this observation, AMPK protein was absent in both CD4^+^ and CD8^+^ T cells, but expressed equivalently in other immune cells in KO mice as compared to WT mice (Figure 1B).

To determine whether AMPK deficiency impacts T cell development, we analyzed the phenotype of lymphocytes in various immune organs. The ratio of T cells to B cells in the peripheral blood (PB), spleen and lymph nodes (LN) was the same between WT and KO mice (Supplementary Figure 1B). Importantly, the ratio of CD4 to CD8 was also equivalent in the thymus, PB, spleen and LNs between the two strains (Figure 1C), and there were no discernible alterations in the percentage of natural regulatory T cells (Tregs) in above organs (Figure 1D). In addition, cell activation markers, including CD25, CD69, CD44 and CD62L, either on CD4^+^ T cells (Figure 1E) or on CD8^+^ T cells (Figure 1F) exhibited similar expression patterns between WT and KO mice. All those observations suggest that depletion of AMPK has a minimal effect on T cell development and activation in naïve mice.

### AMPK specific deletion in T cells promotes tumor growth in mice

**Figure 2:**
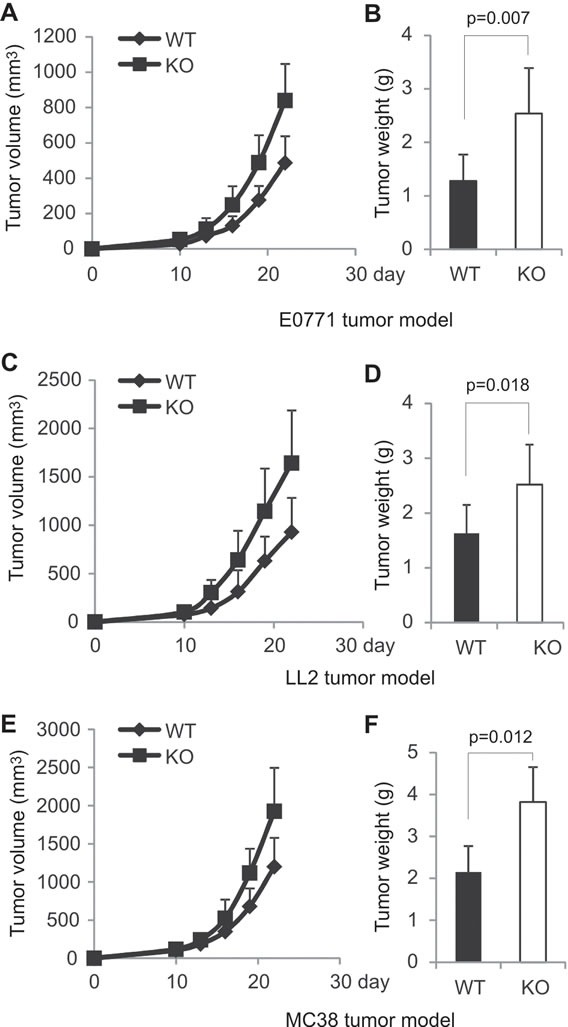
AMPK deficiency in T cells promotes tumor growth in mice A, E0771 cells (0.2×10^6^) were orthotopically injected into the mammary pad of AMPK KO and WT mice (n=9/group). Tumor growth was measured at 3-day intervals. B, weight of E0771 tumor mass was analyzed on day 22 after E0771 cell implantation in mice. LL2 (0.2×10^6^) (C) and MC38 (0.2×10^6^) (E) cells were subcutaneously injected into the right flank of AMPK KO and WT mice (n=9/group), respectively, and tumor growth was measured at 3-day intervals. Weight of LL2 tumors (D) and MC38 tumors (F) was analyzed on day 22 after tumor cell injection. Data shown are representative of at least two independent experiments.

To investigate whether AMPK specific-deletion in T cells impacts tumor growth, we orthotopically injected mammary tumor cells E0771 into the mammary fat pad of KO mice and their WT littermates as previously described [28]. Tumor growth was monitored over a 4-week period after tumor implantation. Although mammary tumors were able to grow in both strains, tumor volume in AMPK KO mice was 2 fold larger as compared to tumors in WT mice (Figure 2A). Average tumor weight in KO mice was also 2 fold greater than that in WT mice (Figure 2B). As AMPK was only deficient in T cells, but not other populations, these results suggest that AMPK expression in T cells is critical in T cell-mediated suppression of tumor growth. To further confirm these observations, we injected other tumor cells originated from different tissues, such as LL2 (Lewis lung carcinoma) and MC38 (colon carcinoma), into these mice and monitored their growth. Consistently, tumor volume and tumor weight of all tested tumors were significantly higher in KO mice than in WT mice (Figure 2C-2F), suggesting a general anti-tumor mechanism by which T cells employ AMPK during tumor development.

### Analysis of T cell presence and function in tumors

**Figure 3:**
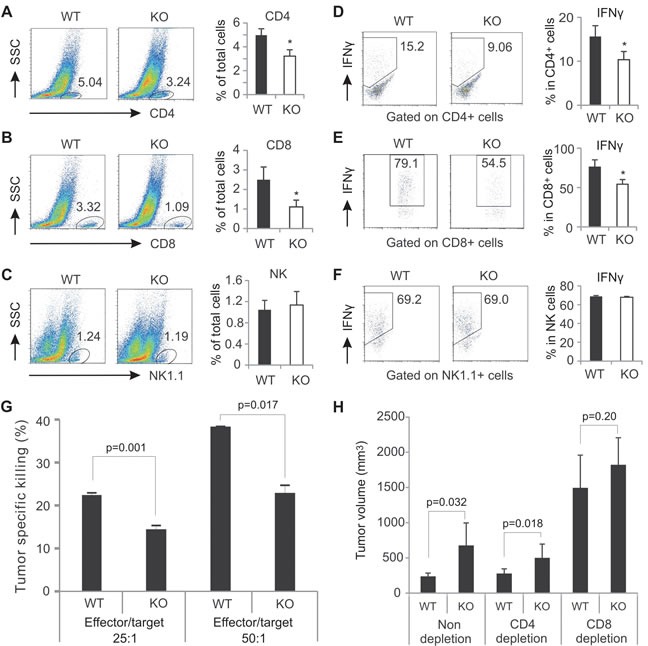
AMPK deficiency decreases T cell presence and function in the tumor stroma Flow cytometric analyses of tumor infiltrating CD4^+^ T cell (A), CD8^+^ T cells (B), and NK cells (C) in E0771 tumors. The percentage of each population was shown in the right panel. Intracellular staining for IFNγ production in CD4^+^ T cells (D), CD8^+^ T cells (E) and NK cells (F) in the tumor stroma after PMA(10ng/ml)/ionomycin (500ng/ml) stimulation for 6 hours *in vitro*. The average of IFNγ^+^-cell percentage in each population was shown in the right panel. G, target cells (E0771) were labeled with CFSE and cocultured with effector cells (collected from dLNs of E0771-tumor bearing mice) at the indicated ratio. The percentage of specific tumor killing was analyzed by flow cytometry. H, measurement of tumor size 19 days after E0771 cell implantation in WT and AMPK KO mice with or without depletion of CD4^+^ cells, or CD8^+^ cells (*, p<0.05 as compared to WT controls).

As AMPK was specifically deleted in CD4^+^ and CD8^+^ T cells, we focused on analyzing T cell presence and functions in the tumor stroma. We first measured T cell presence in tumors. CD4^+^ T cells and CD8^+^ T cells accounted for 5% and 3%, respectively, in E0771 tumors from WT mice. In contrast, they only accounted for 3% and 1% in tumors from KO mice (Figure 3A, 3B). Interestingly, when we compared NK cell infiltration in both tumors, the percentage of NK cells showed no difference between WT and KO mice (Figure 3C). These results indicate that AMPK specific deletion in T cells only lowers the percentage of T cells, but not other immune cells in tumors. We next analyzed T cell functions in tumors. We found that most tumor-infiltrating CD4^+^ T cells were Foxp3^+^ Tregs (around 70% in E0771 tumors), and there were very few Th17 and Th2 cells in the tumor stroma. Interestingly, AMPK deficiency seemed to have minimal influence on the expression of Foxp3, IL-17 and IL-4 in the tumor microenvironment (Supplementary Figure 2A, 2B). Notably, there were about 15% of tumor-infiltrating CD4^+^ T cells expressing IFNγ in WT mice whereas there were only 9% of them in KO mice (Figure 3D), suggesting that AMPK deficiency may impair IFNγ production in tumors. When we analyzed tumor-infiltrating CD8^+^ T cells, we found that majority of the population produced IFNγ (~80%) and Granzyme B (~70%) in the tumor stroma of WT mice. In contrast, these numbers declined to ~55% and ~40%, respectively, when AMPK was absent (Figure 3E, Supplementary Figure 2C). Moreover, we analyzed IFNγ expression in tumor-infiltrating NK cells and found no differences between the two strains (Figure 3F), further suggesting that AMPK expression is indispensable for the optimal production of IFNγ in tumor infiltrating T cells. Taken together, these data indicate that AMPK conditional deletion in T cells specifically affects the presence and IFNγ production of both CD4^+^ and CD8^+^ T cells in tumors.

### CD8^+^ T cells are the major effector cells mediating the anti-tumor effect of AMPK

To investigated whether AMPK deficiency in T cells impacts their specific tumor killing activity, effector lymphocytes from E0771 tumor-bearing WT and KO mice were cocultured with CSFE-labeled E0771 cells. We found that lymphocytes from WT mice displayed significantly higher tumor killing activity than those from AMPK deficient mice (Figure 3G). To further determine which population of T cells is more important in controlling AMPK-mediated anti-tumor effect, as previously described [28], we depleted the CD4^+^ or CD8^+^ population with the respective neutralizing antibodies in mice (Supplementary Figure 2D), and compared tumor growth among depletion and non-depletion groups. As shown in Figure 3H, depletion of the CD4^+^ population showed no impact on E0771 tumor growth in both WT and KO mice as compared to non-depleted groups. However, depletion of the CD8^+^ population remarkably accelerated tumor growth in both strains. More importantly, the difference of the tumor growth in non-depleted groups was diminished when CD8^+^ T cells were absent. These data not only confirm the pivotal role of CD8^+^ T cells in controlling tumor growth, but also establish the CD8^+^ T cell population as the major effector cells mediating the anti-tumor effect of AMPK.

### Deficiency of AMPK impairs anti-tumor functions of CD8^+^ T cells

**Figure 4:**
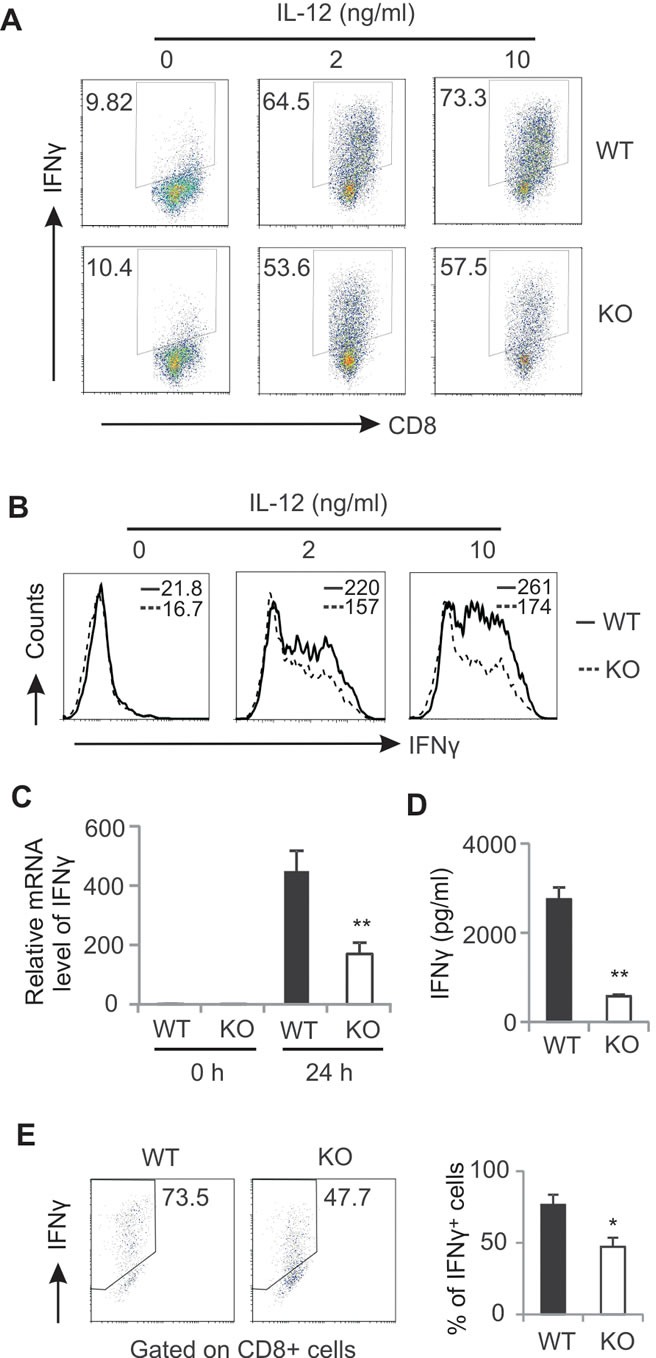
AMPK deficiency impairs CD8 T cell activation A, sorted naïve CD8^+^ T cells were cultured in anti-CD3/CD28-coated wells with indicated concentrations of IL-12 for 72 hours. Production of IFNγ in these cells was analyzed by intracellular staining. The mean fluorescent intensity of IFNγ in these differentiated cells is shown in panel B. Total splenic CD8^+^ T cells were stimulated with anti-CD3/CD28 for 24h. Relative mRNA levels of IFNγ were measured by real-time RT-PCR C, Protein levels of IFNγ in the supernatants were measured by ELISA D,. Expression of IFNγ in CD8^+^ T cells stimulated with anti-CD3/CD28 for 48h was measured by intracellular flow staining. The average percentage was shown in the right panel (E) (*, p<0.05; **, p<0.01). Data shown are representative of at least 3 experiments.

Given the impaired anti-tumor function of AMPK deficient CD8^+^ T cells in tumors, we next investigated the role of AMPK in regulation of anti-tumor functions of CD8^+^ T cells *in vitro*. Naïve CD8^+^ T cells were purified and differentiated into cytotoxic T cells with the stimulation of anti-CD3/CD28 and IL-12. The expression of IFNγ was determined by intracellular staining. Strikingly, both the percentage and intensity of IFNγ expression in AMPK deficient CD8^+^ T cells were significantly lower than those in AMPK sufficient population (Figure 4A, 4B). When total CD8^+^ T cells isolated from the spleen of WT and KO mice were stimulated with anti-CD3/CD28 antibodies, we demonstrated that production of IFNγ in CD8^+^ T cells was also significantly reduced in the absence of AMPK as determined by both mRNA levels and protein levels (Figure 4C-4E). Besides TCR activation of CD8^+^ T cells, we also activated T cells from the spleen and LNs with PMA/Ionomycin and confirmed that the percentage of IFNγ positive population was significantly decreased in CD8^+^ T cells from KO mice as compared to WT mice (Supplementary Figure 3A, 3B). IFNγ levels in the cultural supernatants were also reduced in the absence of AMPK (Supplementary Figure 3C, 3D). In agreement with above *in vivo* observation, these *in vitro* data substantiated that AMPK activation is indispensable in promoting the anti-tumor functions of CD8^+^ T cells.

### AMPK deficiency promotes CD8^+^ T cell death during activation

**Figure 5:**
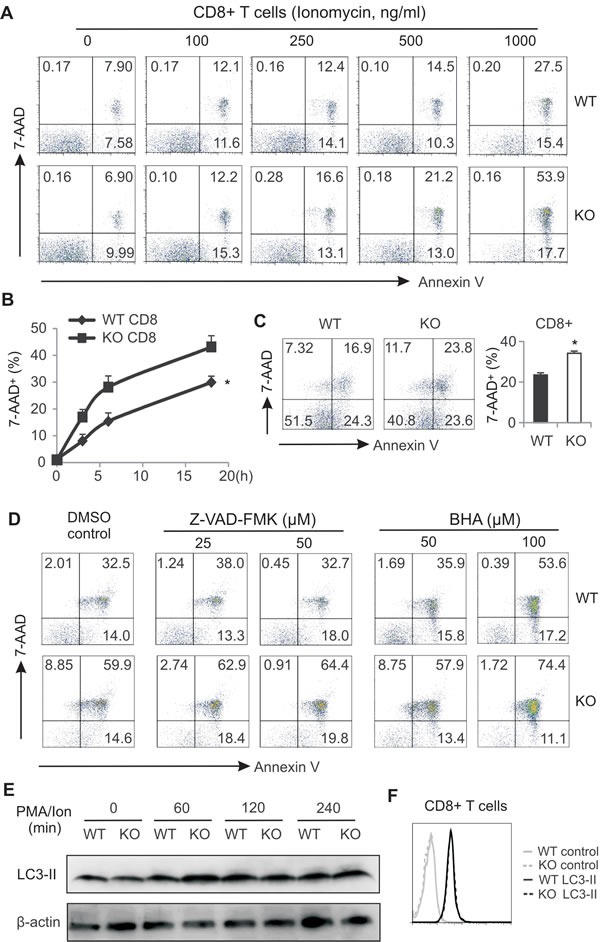
AMPK deficiency promotes CD8 T cell death during activation A, cells from LNs of WT and AMPK KO mice were stimulated with PMA (10ng/ml) plus indicated concentrations of ionomycin for 6 hours. Flow cytometric staining for 7-AAD and Annexin V in CD8^+^ T cells. B, cells from LNs of WT and KO mice were stimulated with PMA (10ng/ml)/ionomycin (500ng/ml) for indicated time periods. CD8^+^ T cell death was analyzed by flow cytometric staining of 7-AAD (*, p<0.05). C, cells from LNs of WT and KO were stimulated with anti-CD3/CD28 for 48h and CD8^+^ T cell death was analyzed by flow cytometric staining. Average cell death was shown in the right panel (*, p<0.05). D, cells from LNs were stimulated with PMA/ionomycin in the presence or absence of indicated concentrations of Z-VAD-FMK or BHA for 6h. Cell death in CD8^+^ T cells was analyzed by flow staining for 7-AAD and Annexin V. E, western blot analysis of LC-3A/3B expression in PMA/ion-stimulated CD8^+^ T cells as indicated time points. F, intracellular staining for LC-3A/3B expression in CD8^+^ T cells after stimulation with PMA/ionomycin for 6h. Data shown are representative of 3 experiments.

As a major metabolic sensor, AMPK can be activated to promote cell survival (12). To dissect how AMPK deficiency impairs CD8^+^ T cell function, we reasoned that AMPK deficiency in T cells may promote cell death due to metabolic demands during activation. Since AMPK can be activated by signals either from Ca^2+^ or from TCR in T lymphocytes [29], we first used ionomycin triggering Ca^2+^ signals in cells from LNs and measured T cell death. We found that ionomycin induced CD8^+^ T cell death in a dose-dependent manner. Particularly, deletion of AMPK increased CD8^+^ T cell death under conditions when ionomycin was present at the high concentrations (500 and 1000ng/ml) (Figure 5A). Similar observations were observed when we analyzed CD4^+^ T cells from WT and KO mice (Supplementary Figure 4A). Of note, there was no difference in cell death between non-T cell populations in LNs from WT and KO mice (Supplementary Figure 4B). To substantiate these observations, we dynamically measured T cell death with the same levels of external stimulation. Again, more CD8^+^ and CD4^+^ T cells died in the absence of AMPK in a time-dependent manner (Figure 5B, Supplementary Figure 4C). In contrast, cell death of non-T cell populations was the same between the two strains (Supplementary Figure 4D). Moreover, when we used TCR triggering of cells from LNs *in vitro*, we observed AMPK deficiency induced more cell death in both CD8^+^ and CD4^+^ populations as compared to AMPK sufficient T cells (Figure 5C, Supplementary Figure 4E). However, cell death exhibited no difference in non-T cell populations where AMPK was not ablated in both WT and KO mice (Supplementary Figure 4F). Altogether, these data clearly demonstrated that AMPK deficiency promotes T cell death in situations where it is activated by different signals.

To determine the cell death modality induced by AMPK deficiency, we noticed that AMPK deficiency did not increase the percentage of Annexin-V single positive apoptotic population in both CD8^+^ and CD4^+^ T cells (Figure 5A and Supplementary Figure 4A). In addition, addition of Z-VAD-FMK, a general caspase inhibitor, exhibited minimal impact on ionomycin-induced CD8^+^ T cell death between WT and KO mice (Figure 5D). These results suggest that apoptotic cell death is not the major mechanism in this process. Furthermore, we tested whether reactive oxygen species (ROS)-mediated cell death is involved in these events [30, 31]. We found that inhibition of ROS by BHA [32] did not diminish the difference of CD8^+^ T cell death between WT and KO mice (Figure 5D). We also measured autophagic activity by examining LC-3B protein levels in activated CD8^+^ T cells. Results from both western blot and intracellular staining showed no major differences in CD8^+^ T cells from WT and KO mice (Figure 5E, 5F). Thus, these data suggest that neither apoptosis- nor autophagy-mediated cell death is the major mechanisms by which AMPK deficiency promotes T cell death.

### Loss of viability in AMPK-deficient T cells is mediated by elevated phosphatase activity

**Figure 6:**
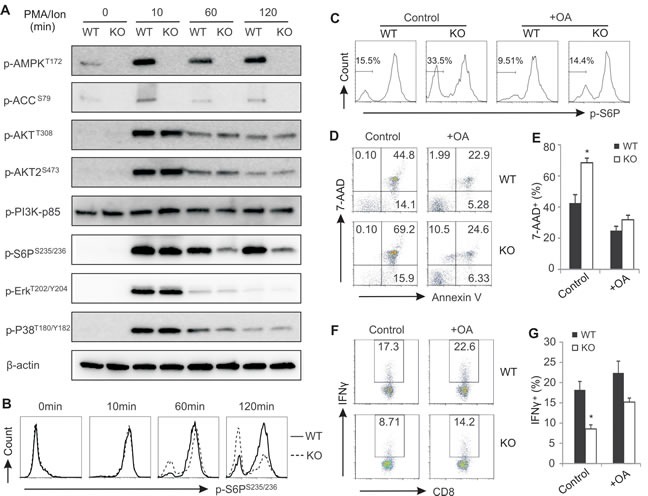
Enhanced phosphatase activity in AMPK deficient CD8 T cells promotes cell death during activation A, purified CD8^+^ T cells from LNs were stimulated with PMA/ionomycin for indicated time periods. Cells were lyzed and analyzed for activation of AMPK, ACC, AKT, AKT2, PI3K, S6P, ERK, p38 by western blot. β-actin was used as a loading control. B, intracellular staining for phosphorylation of S6P in CD8^+^ T cells with PMA/ionomycin stimulation for indicated time periods. C, intracellular analysis of phosphorylation of S6P in PMA/ionomycin-activated CD8^+^ T cells in the presence or absence of okadaic acid (OA, 0.5μM). D, flow cytometric analysis of PMA/ionomycin-activated CD8^+^ T cell death in the presence or absence of OA (1μM). Average percentage of dead CD8^+^ T cells was shown in panel E. Intracellular staining for IFNγ expression in PMA/ionomycin-activated CD8^+^ T cells in the presence or absence of OA (1μM). Average percentage of IFNγ^+^ cells was shown panel G. Data shown are representative of 3 experiments.

To dissect the molecular mechanisms by which AMPK deficiency promotes T cell death during activation, we analyzed major AMPK-mediated signaling pathways in CD8^+^ T cells from AMPK KO and WT mice. As shown in Figure 6A, ablation of AMPK in CD8^+^ T cells abrogated phosphorylation of AMPK and its substrate acetyl CoA carboxylase (ACC). AMPK deficiency seemed to have no discernible impact on the phosphorylation of AKT1, AKT2, PI3K, P38, ERK in activated T cells. However, we noticed that phosphorylation of S6 ribosomal protein (S6P) (Ser235/236) was not sustained in activated CD8^+^ T cells when AMPK was absent. To confirm this observation, we performed intracellular staining by flow cytometry to dynamically track the phosphorylation levels of S6P in specific lymphocyte populations. Compared to unstimulated lymphocytes from LNs, ionomycin stimulation induced rapid phosphorylation of S6P (Ser 235/236) in CD8^+^ T cells from both WT and KO mice (10 min), suggesting that protein kinase activity of S6P was not affected by AMPK deficiency. On the contrary, dephosphorylation of S6P was obviously enhanced in AMPK-deficient CD8^+^ or CD4^+^ T cells at 60min and 120min after ionomycin stimulation (Figure 6B, Supplementary Figure 5A). Of note, under the same condition dephosphorylation levels of S6P remained the same for non-T cell populations (Supplementary Figure 5B). These data suggest that, instead of affecting protein kinase activity, AMPK deficiency may enhance protein phosphatase activity in T cells.

We next used okadaic acid (OA), a specific inhibitor of serine/threonine protein phosphatases [33-35], to inhibit protein phosphatase activity in activated T cells and observed whether AMPK-deficiency-promoted dephosphoryation and cell death can be reversed. Indeed, addition of OA obviously inhibited S6P dephosphorylation in AMPK deficient CD8^+^ and CD4^+^ T cells while leaving non-T cell populations unaffected (Figure 6C, Supplementary Figure 5C). Very strikingly, okadaic acid not only inhibited CD8^+^ T cell death in both WT and KO mice, but also diminished the difference of T cell death between WT and KO mice (Figure 6D, 6E). Moreover, addition of okadaic acid also increased the production of IFNγ in activated CD8^+^ T cells, and decreased the discrepancy of IFNγ production in T cells from KO and WT mice as well (Figure 6F, 6G). As fatty acids have been shown to regulate protein phosphatase activity [36, 37], we measured fatty acid contents in CD8^+^ T cells from WT and KO mice. Interestingly, AMPK deficiency increased the levels of fatty acids, including palmitic acid, myristic acid and DHA, in T cells (Supplementary Figure 5D), which may promote the activity of protein phosphatases in AMPK deficient T cells. Altogether, these data suggest that increased phosphatase activity in AMPK-deficient T cells is the primary cause of the increased T cell death and impaired T function during activation.

### AMPK deficiency increases cell death in tumor-bearing mice

**Figure 7:**
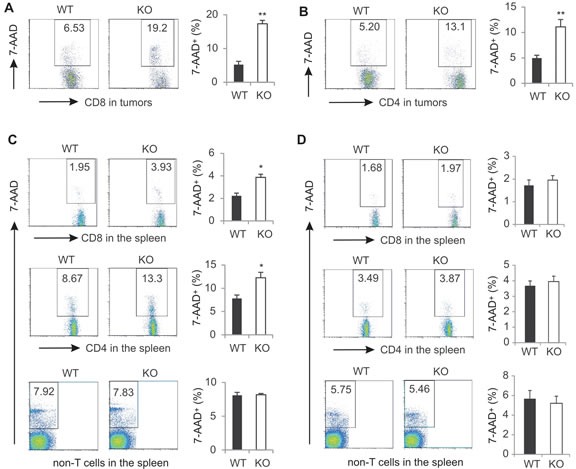
AMPK deficiency increases T cell death in tumor-bearing mice Singe cells were prepared from E0771 tumors from WT and KO mice. Cell death of tumor infiltrating CD8^+^ (A) or CD4^+^ T cells (B) was analyzed by 7-AAD staining. C, splenocytes from tumor-bearing mice were analyzed for cell death in CD8^+^ cells, CD4^+^ T cells, or non-T cell populations. D, splenocytes from naïve mice were analyzed for cell death in CD8^+^ cells, CD4^+^ T cells, or non-T cell populations. (*, p<0.05; **, p<0.01).

On the basis of the above *in vitro* results, we reasoned that the decreased percentage and function of T cells in tumors from AMPK KO mice (Figure 3) may be due to the increased cell death induced by AMPK deficiency. To that end, we directly measured tumor-infiltrating T cell death in tumor-bearing mice without any *in vitro* stimulation. Indeed, we found that ~20% of the CD8^+^ T cells died in the tumor stroma of AMPK KO mice, whereas only ~6% CD8^+^ T cells died in tumors of WT mice (Figure 7A). Similarly, tumors from AMPK KO mice exhibited 2 fold increase of CD4^+^ T cell death as compared to tumors from WT mice (Figure 7B). Furthermore, splenic T-cell populations from AMPK KO tumor-bearing mice also displayed enhanced cell death demonstrating that the loss of viability was not limited to the tumor microenvironment (Figure 7C). In contrast, the non-T populations exhibited a similar death ratio between WT and KO, further indicating the essential role of AMPK in mediating the observed effect (Figure 7C). It is worth noting that splenic cell death in naïve mice was much lower when compared to tumor-bearing mice, and AMPK deficiency showed no effect on T cell death in these mice (Figure 7D). In addition, we also measured the expression of CCR4, CCR5 and CXCR3 receptors on tumor infiltrating CD8^+^ and CD4^+^ T cells from both WT and KO mice and found AMPK deficiency had no overt impact on the expression of these major chemokine receptors (Supplementary Figure 6A-6D). Consistently, expression levels of these chemokine receptors on T cells from peripheral lymph organs were also similar in WT and KO mice (Supplementary Figure 6E-6H). Taken together, these data suggest that AMPK deficiency does not affect T cell recruitment, but promotes T cell death, thereby leading to reduced percentage and function of effector T cells in the tumor microenvironment.

## DISCUSSION

Carcinogenesis is a multistep complex process which depends not only on intrinsic genetic mutations in cancer cells but also on extrinsic effects of immune cells. While many studies have investigated the contribution of AMPK on tumorigenesis by focusing on regulation of tumor cell activities, how AMPK regulates immune cell function during tumor development remains largely unknown. We and others have shown that AMPK, as a major cellular energy sensor, regulates critical functional properties of immune cells, including macrophage polarization and memory T cell differentiation [38-40]. Here we provide new evidence that AMPK is critical in tumor immunosurveillance. Although deletion of AMPK in T cells seems to have no effect on their development, it impairs the anti-tumor functions of T cells, in particular CD8^+^ T cells, resulting in increased tumor growth (Figure 1 and 2). We further find that the loss of antitumor activity is associated with reduced viability and cytotoxic function of activated CD8^+^ T cells (Figure 3 and 4). Finally, we demonstrate that protein phosphatases are the main mediators of cell death in AMPK KO T cells as inhibition of phosphatase activity by OA successfully restores T cell survival and function (Figure 5, 6, 7). Altogether, the data presented here reveal a novel mechanism by which AMPK regulates protein phosphatase activity in control of survival and cytotoxic function of CD8^+^ T cells, thus enhancing their tumor immunosurveillance properties.

Tumors are infiltrated with a variety of types of immune cells that may play dual roles during tumor development and progression [41, 42]. Considering the complexity of immune responses to tumors, we conditionally deleted AMPKα1 in T cells and focused on exploration of AMPK influence on T cell control of tumor growth. Using different syngeneic tumor models we consistently observed an increased tumor growth in the KO mice, indicating that AMPK is essential to T cell-mediated anti-tumor activity (Figure 2). In agreement with our previous studies in other animal models [28], depletion of CD4^+^ T cells in these mice did not affect tumor growth in either WT or AMPK KO mice, suggesting that the AMPK-associated changes in tumor growth were not mediated by CD4^+^ T cells. It should be noted that we did observe the reduced IFNγ production in AMPK KO CD4^+^ T cells in comparison to their WT cohort, however, concomitant loss of the Tregs in CD4^+^ T cell-depleted mice likely masked a potential effect on the antitumor activity of CD4^+^ effector T cells [43, 44]. In contrast, when we depleted CD8^+^ T cells in these mice, tumors grew at a faster rate than those in control mice, and more strikingly, AMPK-mediated tumor protection disappeared in CD8^+^ T cell-depleted mice (Figure 3). Thus, our results presented here not only support a critical role of CD8^+^ T cells in protection against tumor growth, but also establish CD8^+^ T cells as the major effector population in executing AMPK-dependent tumor suppression.

Given the fundamental role of AMPK in coordinating cell metabolic balance and survival [45], we demonstrated that ablation of AMPK in CD8^+^ T cells greatly impairs their anti-tumor functions through promoting cell death under both *in vitro* and *in vivo* settings (Figure 5, 6, 7). As cell death is usually associated with three forms of programmed cell death, including apoptosis, autophagic cell death and necrotic cell death [46, 47], we investigated the effector mechanisms by which AMPK deficiency enhances T cell death. Neither Z-VAD-FMK, a pan-caspase chemical inhibitor, nor BHA, a ROS inhibitor, was able to suppress AMPK-enhanced T cell death. In addition, there was no obvious enhancement of autophagosome formation in T cells in either the WT or the KO mice (Figure 5). Although AMPK has been shown to regulate autophagy and apoptosis in different types of cells [48, 49], our data suggest that AMPK deficiency may result in the induction of other mechanisms (i.e. Ca^2+^-increased, ATP-deficiency-induced programmed cell necrosis) to promote T cell death in tumors [50].

Further analysis revealed that during T cell activation, the AMPK-regulated S6P signaling pathway is downregulated in the absence of AMPK. We also showed that the decreased levels of phospho-S6P are not due to the impaired kinase activity, but due to the enhanced activity of protein phosphatases in AMKP deficient T cells (Figure 6). Given the evidence that the enhanced phosphatase activity in AMPK deficient T cells can be inhibited by OA treatment, we speculate that OA-sensitive phosphoprotein phosphatases (PPPs), such as PP1, PP2A, PP2B, etc [51, 52], may contribute to the elevated T cell death during activation. However, the exact type(s) of PPPs elevated by AMPK deficiency in T cells warrants further investigation. Of note, addition of OA did not completely restore IFNγ production in AMPK deficient T cells (Figure 6G), suggesting that PPP-mediated cell death is not fully responsible for the reduced IFNγ production in these cells. Interestingly, we also noticed that NFAT and NFκB activity was reduced in AMPK deficient T cells (data not shown), further implying that, besides regulating protein phosphatase activity, AMPK may impact other regulatory pathways in regulation of T cell functions.

Although AMPK has been shown to play a pivotal role in multiple cellular functions, consistent with other studies [27, 53, 54], we demonstrate that AMPK deficiency is dispensable for T cell development in naïve mice. It is very likely that AMPK is not activated during T cell development. Of note, the exact role of AMPK in T cell functions seems to be inconsistent depending on different experimental settings. For example, AMPK global deficiency was shown to promote IFNγ production in T cells in the MacIver studies while it was dispensable for the same cytokine production in the Mayer studies [53, 54]. In our studies using CD4creAMPK^fl/fl^ mice we showed that AMPK is critical for T cell survival and cytotoxic function. Several reasons could explain these discrepancies. 1) Compared to T cells from AMPK conditional KO mice, T cells from AMPK global KO mice never experience any signals from environmental AMPK activation in antigen presenting cells, exhibiting a different priming status. Thus, they may respond differently to external stimuli. 2) AMPK can be activated by various upstream activators, such as LKB1, CAMKKs, TAK1 and ROS, which may also lead to different responses [2-6]. 3) In some experimental settings, if AMPK is not effectively activated *in vitro*, the requirement of AMPK for T cell functions will not be observed. For instance, our data showing that AMPK deficiency has no impact on the survival of T cells when activated using low concentrations of Ionomycin (Figure 5A and Supplemental Figure 4A) also support this speculation.

In summary, we find that the influence of AMPK deficiency on T cell survival and cytotoxic function appears to be more prominent when AMPK is activated *in vitro* with external stimuli, such as Ca^2+^ or TCR signals, or *in vivo* within the tumor stroma. Therefore, our findings support the notion that AMPK is more critical when cells are under diverse stressful conditions, including cell-autonomous energy imbalance or environmental alterations. Considering that AMPK activity has been associated both with pro- and anti-tumorigenic effects depending on the cell type in which it is induced [55-57], our study sheds further light on the seemingly contradictory role of AMPK in tumor progression by demonstrating a specific requirement for AMPK in sustaining the antitumor activity of tumor-associated T cells.

## METHODS

### Mice and cells

All WT and AMPKα1 mice were bred and maintained in the animal facility of the Hormel Institute in accordance with protocols approved by the Institutional Animal Care and Use Committee (University of Minnesota). Floxed *Prkaα1* mice were from Jackson Lab. CD4-Cre mice were from Taconic. Genotyping of mice was performed by PCR with following primers: CCT GGA AAA TGC TTC TGT CCG TTT G and ACG AAC CTG GTC GAA ATC AGT GCG for Cre, CCC ACC ATC ACT CCA TCT CT and AGC CTG CTT GGC ACA CTT AT for floxed *Prkaα1*. Mouse E0771 cells were from CH3 BioSystems; MC38 and LL2 were gifts from Jun Yan (University of Louisville, KY) and were cultured less than 6 months for experiments. Cells were not further authenticated.

### Syngeneic tumor models

Mammary tumor cells E0771 were orthotopically implanted into the mammary fat pad of AMPK conditional KO mice and their littermate WT mice. Tumors were measured at 3 day intervals with calipers and the volume was calculated by the formula 0.4× (large diameter) × (small diameter)^2^. For depletion of CD4^+^ T cells or CD8^+^ T cells, each mouse was intraperitoneally injected with 250μg anti-CD4 (clone GK1.5, Biolegend) or anti-CD8 (clone 53-6.7, Biolegend) neutralizing mAbs daily for 3 days. After confirmation the depletion of CD4^+^ or CD8^+^ T cells, mice were implanted with E0771 cells as described above. To measure immune cell infiltration and functions in tumor stroma, single cells from solid tumors were prepared by digesting dissected tumors in the enzyme mixture (0.5 mg/ml collagenase A (Roche Diagnostic), 0.2 mg/ml type V hyaluronidase and 0.02 mg/ml DNase I (Sigma-Aldrich) in RPMI 1640 at 37°C for 45 min. The separated cells were washed for further analyses.

### Flow cytometric analysis and cell sorting

Surface and intracellular staining were performed as previously described [28]. Single immune cell populations from the spleen, lymph nodes or tumors were separated with a BD FACSAria II Cell Sorter. Flow cytometric analyses were performed with Flowjo (Tree Star). The following antibodies were used for cell staining: anti-CD3 (clone 145-2C11), anti-CD4 (clone RM4-5), anti-CD8 (clone 53-6.7), anti-CD49b (clone DX5), anti-CD11b(clone M1/70), anti-CD11c (clone HL3), anti-CD19 (clone 1D3), anti-CD25 (clone PC61), anti-CD69 (clone H1.2F3), anti-CD62L (MEL-14), anti-CD44 (clone IM7), anti-Foxp3 (clone FJK-16s), anti-Granzyme B (clone GB11), anti-CCR4 (clone 2G12), anti-CCR5 (clone HM-CCR5), anti-CXCR3 (clone CXCR3-173), NK1.1(clone PK136), anti-F4/80 (clone BM8), anti-Gr-1 (clone RB6-8C5), anti-interferon-γ (IFN-γ, clone XMG1.2), and anti-NK1.1 (clone PK136), CD4 blocking mAb (clone GK1.5), and CD8 blocking mAb (clone 53-6.7).

For detection of phosphorylated S6 proteins, cells from LNs cultured with PMA (10ng/ml) and Ionomycin (500ng/ml) at designated times were immediately fixed with phosflow Lyse/Fix buffer (BD Biosciences) and permeabilized by Phosflow Perm buffer (BD Biosciences). Cells were stained with the Alex488 conjugated antibody for S6P (Ser235,236) (D57.2. 2E; Cell Signaling Technology)

### Quantitative real-time RT-PCR

RNA was extracted from cultured or purified primary cells using RNeasy Mini Kit (Qiagen). cDNA synthesis was performed with QuantiTect Reverse Transcription Kit (Qiagen). Quantitative PCR was performed with SYBR® Green PCR Master Mix using ABI 7500 Real-Time RT-PCR Systems (Applied Biosystems). AMPKα1, AMPKα2, IFNγ, CCR4, CCR5, CXCR3 and β-actin expression was analyzed by QuantiTect primer assays (Qiagen). Results were normalized to β-actin. Relative gene expression was measured using the ΔΔCT approach.

### T cell culture and functional analysis

For CD8^+^ T cell differentiation, naïve CD8^+^ T cells separated by a flow sorter were cultured in plates coated with anti-CD3 antibody (clone 145-2C11,5μg/ml) and anti-CD28 antibody (clone 37.51, 2μg/ml) in the presence of different concentrations of IL-12 (0, 2, 10ng) for 72 hours. Supernatants were collected for ELISA measurements. Cells were harvested for real-time PCR analysis and intracellular staining. For intracellular staining, Golgiplug (BD bioscience) was added to the cultural cells for additional 4 hours before analysis.

For analysis of TCR triggering of T cell activation, total cells or purified T cells from the spleen, LNs or tumors from WT and KO mice were cultured in plate coated anti-CD3 antibody (clone 145-2C11,5μg/ml) and anti-CD28 antibody (clone 37.51, 2μg/ml) for designated times. For analysis of Ca^2+^ triggering of T cell activation, total or purified populations (Stem cell CD4/CD8 T cell purification kits) from above lymphoid organs or tissues were stimulated with PMA(10ng/ml)/ionomycin (500ng/ml) for designated times for further analysis.

### Analysis of T cell death

T cell death was analyzed by flow cytometric staining for Annexin V (BD Bioscience) and 7-AAD (BD Bioscience) in different conditions. For *in vitro* activation, cells from the spleen or LNs were activated either with anti-CD3/CD28 signaling or with PMA/ionomycin stimulation. For treatment with Z-VAD-FMK (R&D Systems), BHA (Sigma), or okadaic acid (sigma), cells from LNs were pretreated with indicated concentrations of Z-VAD-ZMK, BHA or okadaic acid for 30min, then stimulated with PMA(10ng)/Ionomycin (1000ng) for 6h. For assessment of cell death *in vivo*, cells in the spleen or tumors from WT and KO mice were directly stained for Annexin V and 7-AAD without any *in vitro* stimulation.

### Western blotting

CD4^+^, CD8^+^, CD11b^+^ or CD19^+^ cells were sorted by a flow sorter from the spleen of WT and AMPKα1 mice for measurement of AMPKα1 expression. CD8^+^ T cells separated from LNs with designated treatments were lysed in buffers with protease and phosphorylation inhibitors. Protein concentrations were determined by BCA assay (Thermo Scientific). β-actin was used as a loading control. The antibodies of AMPKα1(2532), p-AMPK^T172^(4188), p-ACC^S79^(3661), p-S6^S235/236^(4803), p-Erk^T202/Y204^, p-AKT^T308^(2965), p-AKT^s473^(4060), p-AKT2^s473^(8599), p-P38^T180/Y182^(4551), P-p85^Y458^(4288) were purchased from Cell Signaling Technology. LC3A/3B antibody (PA5-22731) was from Thermo Fisher Scientific.

### ELISA

Mouse IFNγ ELISA kit (Biolegend) was used to measure the level of IFNγ in culture supernatants according to manufacturer's protocol.

### Tumor killing assay

Lymphocytes collected from draining lymph nodes of E0771-tumor bearing mice were used as effector cells. Target tumor cells (E0771) were labeled with 2μM CFSE. Effector and target cells were cocultured for 24 hours in a 96-well v-bottom plate. All cells were stained with 7-AAD and analyzed by flow cytometry. Tumor specific killing was calculated as the difference between total % of killed (7-AAD^+^) and % of spontaneous apoptotic E0771 cells in all CFSE^+^ cells.

### Statistical analysis

Unpaired, two-tailed Student's *t* test was performed for the comparison of results from other different treatments. P value less than 0.05 is considered statistically significant.

## SUPPLEMENTARY MATERIALS FIGURES


