# Supplementary material for: Deficiency of AMPK in CD8+ T cells suppresses their anti-tumor function by inducing protein phosphatase-mediated cell death
Source: Oncotarget. 2015 Mar 9;6(10):7944–58. doi: 10.18632/oncotarget.3501 (PMC4480727; doi:10.18632/oncotarget.3501)
Supplement: Supplementary file 1 [file oncotarget-06-7944-s001.pdf]

Deficiency of AMPK in CD8+ T cells suppresses their anti-tumor function by inducing protein phosphatase-mediated cell death

Supplementary Material

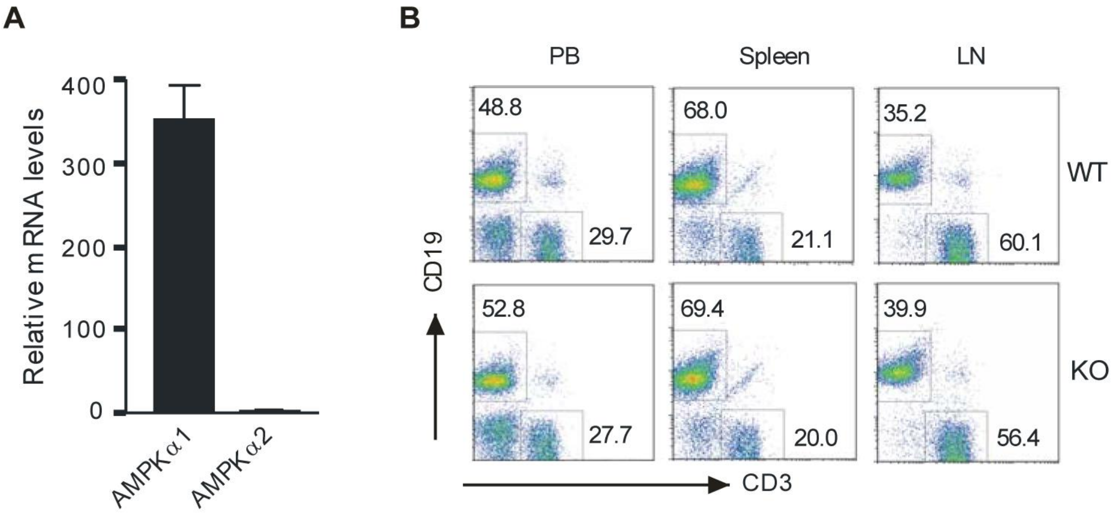

Supplementary Figure 1. Phenotypic analysis of WT and AMPKα1 KO mice. A, real-time RT-PCR analysis of the expression of AMPKα1 and AMPKα2 in T cells from WT mice. **B**, Analysis of the percentage of T cells and B cells in PB, spleen and LNs from WT and KO mice by flow cytometric staining.

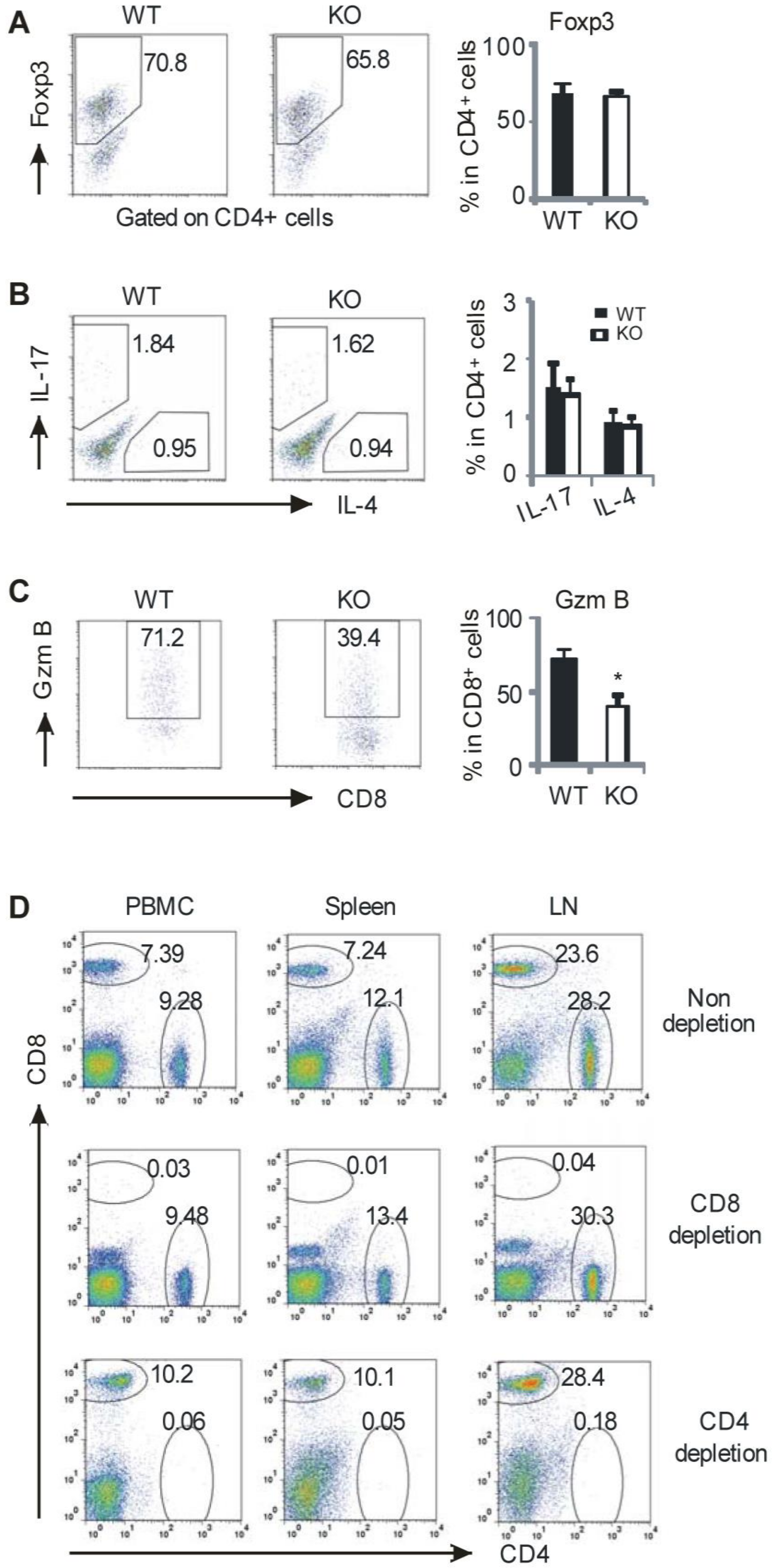

Supplementary Figure 2. Analysis of tumor infiltrating T cell subsets in E0771 tumors. Intracellular staining for expression of Foxp3 (**A**), IL-17 and IL-4 (**B**) in tumor infiltrating CD4+ T cells. The average percentage of each population was shown in the right panel. **C**, intracellular staining for Granzyme (Gzm) B expression in tumor infiltrating $CD8^+$ T cells. The average percentage of Granzyme B^+ population in total $CD8^+$ T cells was shown in the right panel (*, $p<0.05$). **D**, flow cytometric analyses of $CD4^+$ and $CD8^+$ population in peripheral blood (PB), spleen and LNs from mice with or without depletion of $CD8^+$ or $CD4^+$ T cells.

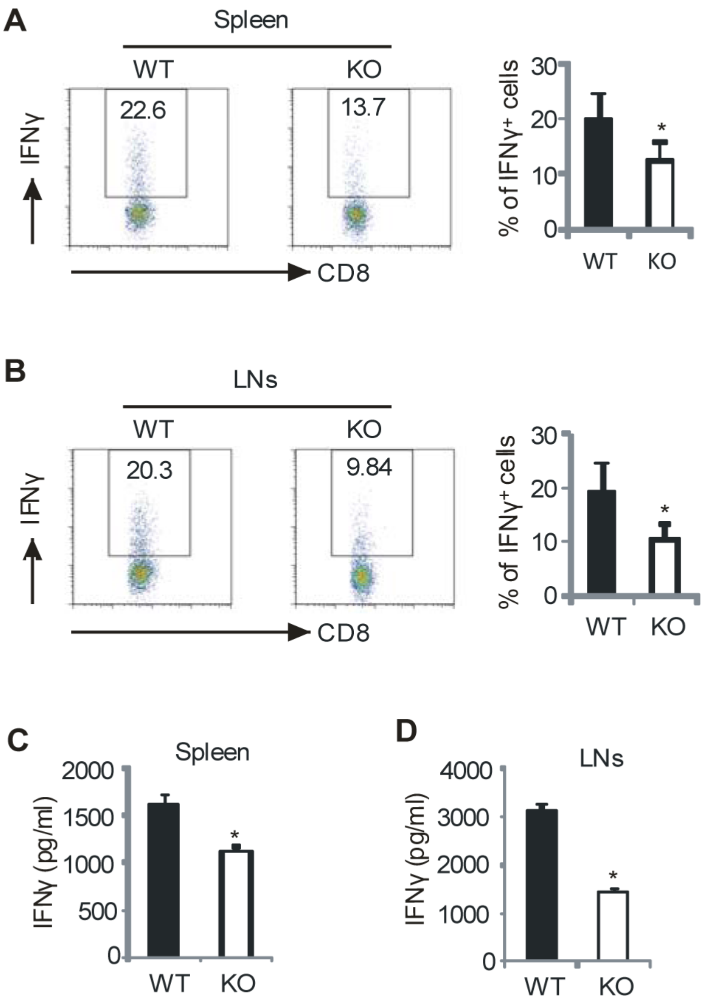

Supplementary Figure 3. Deletion of AMPK reduces IFNγ production in $CD8^+$ T cells. Cells from the spleen and lymph nodes were stimulated *in vitro* with PMA/ionomycin for 6h. IFNγ production in $CD8^+$ T cells from the spleen (**A**) and LNs (**B**) was analyzed by intracellular staining. The average percentage of IFNγ^+ cells was shown in the right panel. IFNγ levels in the cultural supernatants of splenic cells (**C**) and lymphocytes from LNs (**D**) were measured by ELISA (*, $p<0.05$).

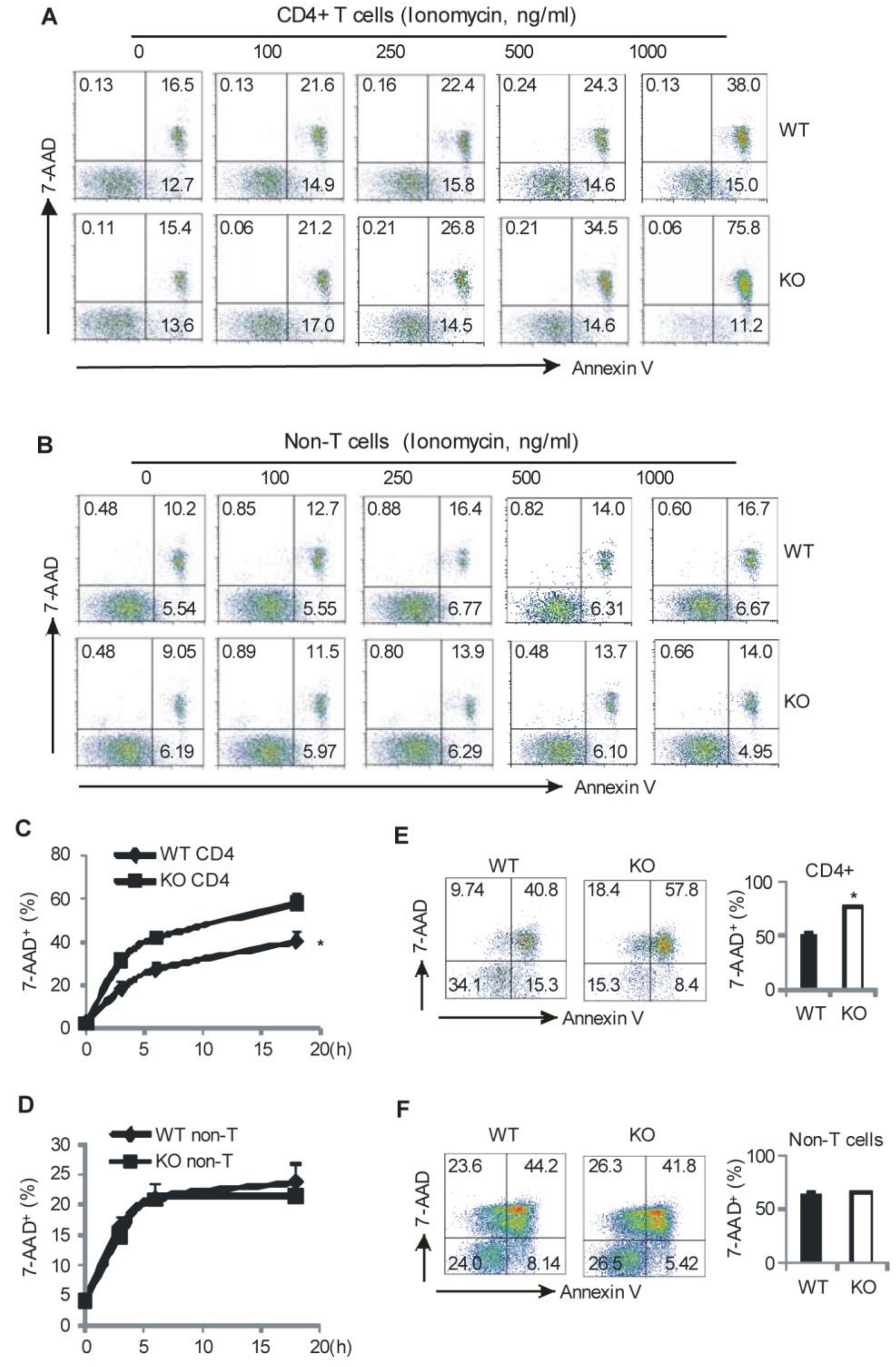

Supplementary Figure 4. AMPK deficiency promotes $CD4^+$ T cell death during activation. Cells for LNs of WT and KO mice were stimulated with PMA (10ng/ml) and indicated concentrations of ionomycin for 6 hours. Flow cytometric staining for 7-AAD and Annexin V in $CD4^+$ T cells (**A**) and in non-T cell populations (**B**). Cells from LNs of WT and KO mice were stimulated with PMA (10ng/ml)/ionomycin (500ng/ml) for indicated time periods. Cell death of $CD4^+$ T cells (**C**) and non-T populations (**D**) was analyzed by flow cytometric staining for 7-AAD. Cells from LNs of WT and KO were stimulated with anti-CD3/CD28 for 48h. Cell death of $CD4^+$ T cells (**E**) and non-T populations (**F**) was analyzed by flow cytometric staining (*, $p<0.05$).

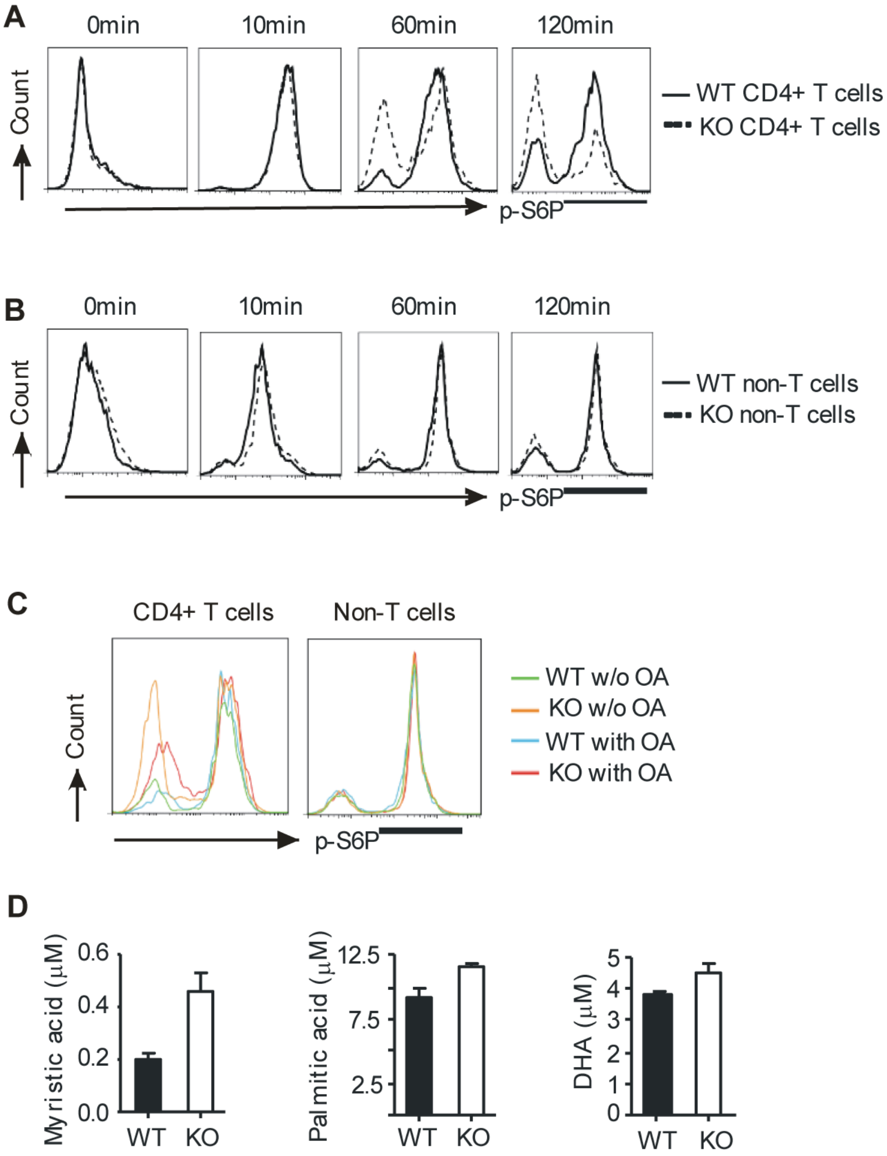

Supplementary Figure 5. Elevated phosphatase activity in AMPK deficient CD4^{+} T cells. **A**, intracellular staining for phosphorylation of S6P in CD4^{+} T cells with PMA/ionomycin stimulation for indicated time periods. **B**, intracellular staining for phosphorylation of S6P in non-T cell population with PMA/ionomycin stimulation for indicated time periods. **C**, intracellular analysis of phosphorylation of S6P in PMA/ionomycin-activated CD4^{+} T cells and non-T cell populations in the presence or absence of okadaic acid (OA, 0.5μM). **D**, Analysis of fatty acids contents in CD8+ T cells from WT and KO mice by lipid mass spectrometry.

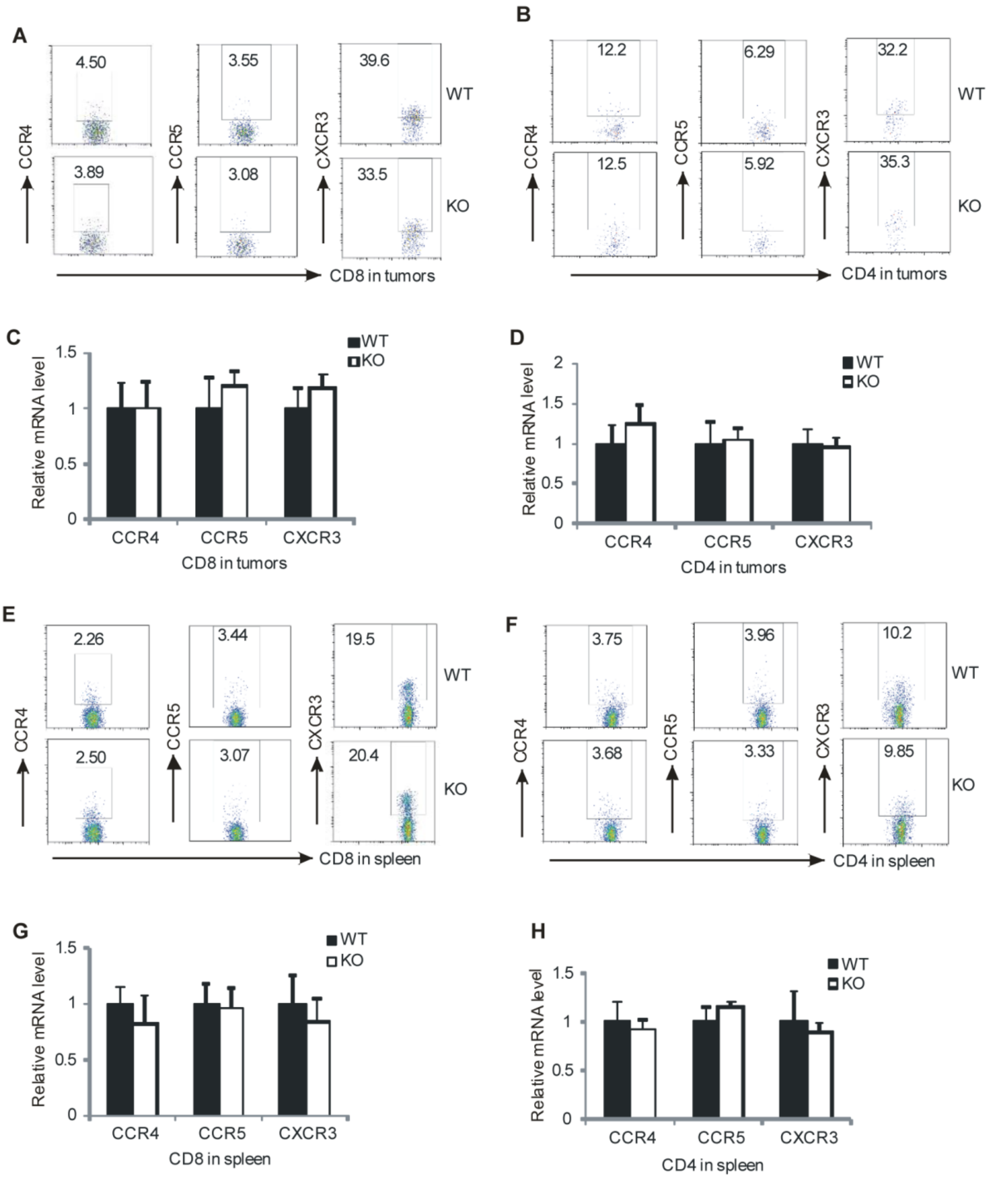

Supplementary Figure 6. AMPK deficiency has no impact on expression of chemokine receptors on T cells. A, B, flow cytometric staining of the expression of CCR4, CCR5, CXCR3 on $CD8^+$ (**A**) or on $CD4^+$ T cells (**B**) from the tumor stroma of WT and KO mice. **C**, **D**, real-time RT-PCR analysis of CCR4, CCR5 and CXCR3 expression in $CD8^+$ T cells (**C**) or in $CD4^+$ T (**D**) cells separated from tumors. **E, F,** flow cytometric staining of the expression of CCR4, CCR5, CXCR3 on $CD8^+$ (**E**) or on $CD4^+$ T cells (**F**) from the spleen of tumor-bearing WT and KO mice. **G,H**, real-time PCR analysis of CCR4, CCR5 and CXCR3 expression in $CD8^+$ T cells (**G**) or in $CD4^+$ T cells (**H**) cells separated from the spleen of tumor-bearing WT and KO mice.
